# The Effects of TRX Suspension Training Combined with Taurine Supplementation on Body Composition, Glycemic and Lipid Markers in Women with Type 2 Diabetes

**DOI:** 10.3390/nu13113958

**Published:** 2021-11-05

**Authors:** Shohreh Samadpour Masouleh, Reza Bagheri, Damoon Ashtary-Larky, Neda Cheraghloo, Alexei Wong, Omid Yousefi Bilesvar, Katsuhiko Suzuki, Marefat Siahkouhian

**Affiliations:** 1 Department of Sport Physiology, Faculty of Educational Sciences and Psychology, University of Mohaghegh Ardabili, Ardabil 56131-56491, Iran; shohrehsamadpour@gmail.com (S.S.M.); O_Yousefi@ymail.com (O.Y.B.); 2 Department of Exercise Physiology, University of Isfahan, Isfahan 81746-73441, Iran; will.fivb@yahoo.com; 3 Nutrition and Metabolic Diseases Research Center, Ahvaz Jundishapur University of Medical Sciences, Ahvaz 61357-15794, Iran; damoon_ashtary@yahoo.com; 4 Department of Epidemiology and Biostatistics, School of Public Health, Tehran University of Medical Sciences, Tehran 14176-13151, Iran; neda_cheraghloo721@yahoo.com; 5 Department of Health and Human Performance, Marymount University, Arlington, VA 22207, USA; awong@marymount.edu; 6 Faculty of Sport Sciences, Waseda University, Mikajima, 2-579-15, Tokorozawa 359-1192, Japan

**Keywords:** taurine, body composition, TRX, nutritional supplements, type 2 diabetes

## Abstract

Background: We aimed to investigate the effects of an 8-week total-body resistance exercise (TRX) suspension training intervention combined with taurine supplementation on body composition, blood glucose, and lipid markers in T2D females. Methods: Forty T2D middle-aged females (age: 53 ± 5 years, body mass = 84.3 ± 5.1 kg) were randomly assigned to four groups, TRX suspension training + placebo (TP; *n* = 10), TRX suspension training + taurine supplementation (TT; *n* = 10), taurine supplementation (T; *n* = 10), or control (C; *n* = 10). Body composition (body mass, body mass index (BMI), body fat percentage (BFP)), blood glucose (fasting blood sugar (FBS)), hemoglobin A1c (HbA1c), Insulin, and Insulin resistance (HOMA-IR), and lipid markers (low-density lipoprotein (LDL), high-density lipoprotein (HDL), triglyceride (TG), and total cholesterol (TC)) were evaluated prior to and after interventions. Results: All three interventions significantly decreased body mass, BMI, and BFP with no changes between them for body mass and BMI; however, BFP changes in the TT group were significantly greater than all other groups. FBS was significantly reduced in TP and TT. Insulin concentrations’ decrement were significantly greater in all experimental groups compared to C; however, no between group differences were observed between TT, TP, and T. In regards to HOMA-IR, decreases in TT were significantly greater than all other groups TG, HbA1c, and LDL were reduced following all interventions. HDL values significantly increased only in the TT group, while TC significantly decreased in TP and TT groups. Changes in HbA1c, TG, HDL, and TC were significantly greater in the TT compared to all other groups. Conclusions: TRX training improved glycemic and lipid profiles, while taurine supplementation alone failed to show hypoglycemic and hypolipidemic properties. Notably, the synergic effects of TRX training and taurine supplementation were shown in HbA1c, HOMA-IR, TG, TC, HDL, and BFP changes. Our outcomes suggest that TRX training + taurine supplementation may be an effective adjuvant therapy in individuals with T2D.

## 1. Introduction

Type 2 diabetes (T2D) is related to a host of health threats such as metabolic syndrome, cardiovascular disease, and mental disorders [1,2]. These complications have higher incidence and mortality rates in women compared to men [3,4], even though women are more proactive about managing T2D [5], suggesting that impaired estrogen concentration with T2D may reduce the protective effect of this female hormone in negative physiological processes [6]. Research has extensively indicated that obesity resulting from sedentarism and extreme dietary intakes can accelerate the development of T2D and its complications [7,8,9]. Exercise interventions are widely recommended to combat T2D among various populations [10]. Indeed, resistance training (RT) programs have gained substantial popularity as an efficient tool for ameliorating insulin sensitivity and glycemic control in individuals with this condition [11,12]. As a newly introduced form of RT, total body resistance exercise (TRX) involves suspension training, which allows for the performance of single and multi-joint exercises using body weight and gravity as resistance. This training modality was originally developed for therapy and rehabilitation, but due to its gentle nature and an individual’s ability to simply adjust body position to increase or reduce resistance, it has become popular for different cohorts that are unable to perform intense RT. This may be relevant for middle-aged and older women with T2D, as musculoskeletal discomfort and physical limitations are important barriers leading to high adherence to conventional RT programs in this population [13,14]. Previous studies have shown the positive effects of TRX training on cardiometabolic and fitness parameters in non-diabetic populations, including body fatness [15], waist circumference [15], blood pressure [15], HDL [16] and liver enzyme concentrations [17], as well as muscle mass and strength [18,19,20]. Although studies evaluating TRX in individuals with T2D are lacking, recent research showed that this training modality improved fasting blood sugar (FBS) and insulin concentrations in women with polycystic ovary syndrome (PCOS) [21]. Because hyperinsulinemia is common in PCOS, these findings indicate that TRX may improve glycemic profiles in women with T2D.

In addition to exercise interventions, several nutritional strategies such as amino acid supplementations have been recommended to treat T2D. Namely, taurine is a conditionally semi-essential amino sulphonic acid derived from methionine and cysteine metabolism [22]. This amino acid protects β-cells against destruction by streptozotocin in a dose-dependent fashion [23]. Research shows that plasma and platelet taurine in diabetes mellitus are decreased, and platelets in non-insulin-dependent diabetes mellitus show alterations in taurine transport [24]. In addition, taurine supplementation combined with a low-protein diet has been shown to normalize insulin secretion from islets in rats [25]. Most of the studies related to taurine supplementation and its effects on diabetic outcomes were conducted in animal models, which showed its effectiveness in improving glycemic markers (e.g., FBS, insulin, hemoglobin A1C, etc.) [26,27,28,29,30]. These beneficial effects have also recently been reported in humans, as Maleki et al. showed an improvement in glycemic and lipid markers in 23 Iranian patients with T2D after eight weeks of taurine supplementation [31]. Although there is evidence of the favorable impact of both taurine supplementation and TRX training on glycemic and lipid profiles, the effect of the combination of these two interventions has not been determined. The addition of a nutritional approach to TRX training may be essential to positively affect glycemic and lipid profiles in those with T2D, and potentially establish an additive effect not achieved by each intervention alone. Therefore, the primary purpose of this investigation was to assess the effectiveness of an 8-week TRX training intervention combined with taurine supplementation on glycemic and lipid profiles in women with T2D. A secondary purpose was to evaluate body composition, as this might help explain some potential favorable changes in metabolic parameters.

## 2. Materials and Methods

### 2.1. Participants

**Figure 1:**
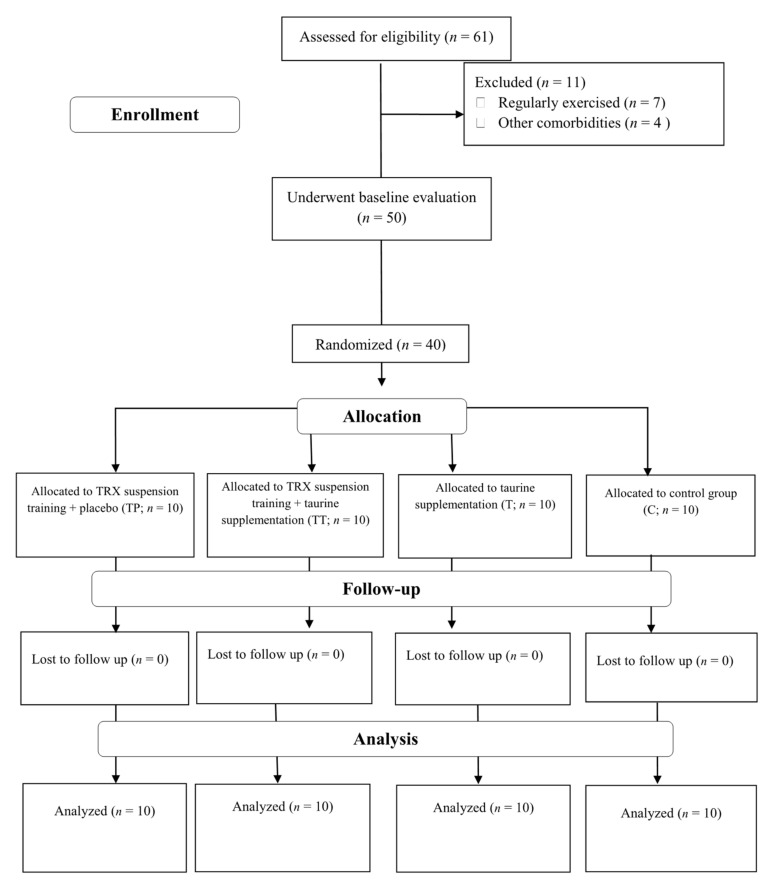
Participants Flow Diagram. Abbreviations: T, taurine supplementation; TP, TRX suspension training + placebo; TT, TRX suspension training + taurine supplementation; C, control.

A participant flow diagram is depicted in Figure 1, and their characteristics are presented in Table 1. Forty sedentary middle-aged T2D females from Rasht, Iran, participated in this investigation. Inclusion criteria comprised of: having T2D at least for six months prior to the study, no history of cardiovascular disease (including hypertension), no smoking, no usage of any dietary supplements at least for six months prior to the study, no change in drugs for FBS reduction, and between the age ranges of 40–60 years. All participants were on medication: metformin (500 mg, two times per day with meals) and glibenclamide (5 to 10 mg in the morning in a fasted state). Exclusion criteria included: voluntary withdrawal of the participants from the research, enrolling in any other exercises other than training protocol of this study, significant musculoskeletal deformity (i.e., amputation, scoliosis, abnormality of range of motion (ROM)), injuries or pain that limits exercise, the presence of severe cardiovascular diseases, angina-equivalent symptoms (i.e., nausea, diaphoresis, and shortness of breath with exercise), changes in medications or intake of any dietary supplement not assigned by researchers. All our participants provided their written informed consent, and the ethics committee of the University of Mohaghegh Ardabili, Ardabil, Iran, approved the study protocol. The study was registered in the sports sciences research institute of Iran (IR.SSRI.REC.1400.971) and carried out in accordance with the Declaration of Helsinki.

Participants were randomly assigned to four groups as follows: TRX suspension training + placebo (TP; *n* = 10), TRX suspension training + taurine supplementation (TT; *n* = 10), taurine supplementation (T; *n* = 10), or control (C; *n* = 10). All participants were fully familiarized with testing and experimental procedures prior to baseline measurements. Measurements were recorded at baseline and after 8 weeks (post) at the same time (within ~1 h) in the morning. Post measurements took place approximately 72 h after the last exercise session in order to minimize potential acute influences of TRX on our outcomes. Participants were instructed to avoid altering their usual lifestyle and dietary habits during the investigation.

### 2.2. Anthropometry and Body Composition

We instructed our participants to fast for 12 h (overnight fast, with at least 8 h of sleep) and refrain from physical activity prior to all measurements. Upon arriving at the laboratory, participants were asked to void completely within 30 min prior to data collection. Participants’ body mass was measured with a digital scale (Seca, Hamburg, Germany) to the nearest 0.1 kg, and stature was measured with a stadiometer (Seca, Hamburg, Germany) to the nearest 0.1 cm. Triceps, subscapular, and abdomen skinfold thicknesses were measured on the body’s right side by a trained investigator using a calibrated caliper (Harpenden, Baty, UK) in a double-blind manner. Two measurements were performed. If the mean differences between measurements were less than 5%, the mean of those two numbers was used to calculate body fat percentage (BFP). However, if the mean of those two numbers was higher than 5%, a third measurement was performed, and the mean of the three numbers was considered to calculate BFP [32]. The Jackson and Pollock equation was used to determine BFP: Body density = 1.0994921 − (0.0009929 × Total thickness of 3 skinfolds (mm)) + (0.0000023 × (Total thickness of 3 skinfolds) ^2^) − (0.0001392 × age), then calculated BF% = (495/body × density) − 450 [33]. The test re-test ICC of skinfold measurements was higher than 0.987. All measurements were collected by the same investigator at baseline and post-testing.

### 2.3. Blood Sampling and Laboratory Analysis

Fasting blood samples (10 mL) were collected from the forearm vein in a resting position. The sampling process was conducted from 8:00 a.m. to 9:00 a.m. for both baseline and post-interventions to validate the outcomes. This avoided any diurnal effects and minimized confounding variables. The samples were collected in vacutainers for analysis, and following centrifugation (1000× *g* for 15 min), serum was removed from the samples and stored at −70 °C for further analyses. The enzyme-linked immunosorbent assay (ELISA) kits (Diaplus Inc., North York, ON, Canada) were used to measure plasma insulin concentrations. FBS, high-density lipoprotein (HDL), low-density lipoprotein (LDL), total cholesterol (TC), triglyceride (TG), and hemoglobin A1c (HbA1c) were measured by the spectrophotometric method (Pars Azmoon Inc., Tehran, Iran) using an auto-analyzer (Hitachi, White Plains, NY, USA). Insulin resistance (HOMA-IR) was measured using the following formula [34,35,36]:

HOMA-IR = (Fasting insulin) ∗ (Fasting glucose)/405

### 2.4. TRX Suspension Training Protocol

A week before initiating the training protocol, participants in both TP and TT groups were familiarized with training protocols and exercise techniques. Training protocols were performed three times per week for 8 weeks. The total training duration per session was 60 min and entailed 45 min of TRX suspension training and light jogging and stretching for a warm-up, and stretching for a cool down. The exercises included: push-ups, chest press, standing rowing, lunges, and squats. The training intensity was controlled (Table 2) based on Borg rating of perceived exertion (RPE). Exercises were performed at RPE 14 to 17 with repetitions ranging from 8 to 12. Our TRX training protocol (including the progression of volume and intensity) is based on prior research [20,21]. All TRX sessions were completed in Rasht under the supervision of certified trainers who were blinded to supplement assignments.

### 2.5. Taurine and Placebo Supplementation

Participants in both TT and T were supplemented by 5 capsules (500 mg) of taurine in the morning and before bed. The timing and dosage of taurine were based on previous study [31]. Participants in the TP and C group consumed a placebo (dextrose filled capsules (500 mg)). The capsules had a similar appearance and were produced by the same laboratory.

### 2.6. Diet

Participants completed dietary logs (2 weekdays and 1 weekend day) at pre-test and immediately after the study. Food items were entered and analyzed (Diet Analysis Plus, v10; Cengage, Boston, MA, USA) to determine changes in total energy (kcal), carbohydrate, fat, and protein over time [37,38].

### 2.7. Statistical Analysis

A priori sample size calculation was performed utilizing the G*Power analysis software [39]. The sample size was based off prior data [21]. Based on an α value of 0.05 and a power (1–β) value of 0.80, the analysis showed that a total sample size of at least thirty-two participants (*n* = 8 per group) was necessary to have sufficient power to detect significant changes in FBS and insulin concentrations. Data were assessed for normality using the Shapiro–Wilk test. Descriptive analysis was represented using mean ± standard deviation (SD). An analysis of variance (ANOVA) was performed on mean variables between groups. The Tukey’s HSD (honestly significant difference) test was used to determine whether the difference between the two groups was statistically significant. In order to compare the mean values of a pair of groups, the Bonferroni test was applied. Two variables were compared using the paired *t*-test on the same subject. Based on the analysis of covariance (ANCOVA), it was determined whether the means of variables in the post-test were the same as the means across levels of group controlling for variables in the pre-test. The statistical analyses were conducted in SPSS (v26). All figures were made using Graphpad Prism software (8.4.3). *p*-values less than 0.05 were considered statistically significant.

## 3. Results

### 3.1. Study Compliance, Diet, and Body Composition

The compliance with training and supplement was 100%. There were no significant baseline differences between groups for any body composition measures. All three intervention groups had significant (*p* < 0.05) decreases in body mass (T = −2.7 kg (95% confidence interval (CI) = −1.6 to −3.7; *p* < 0.001), TP = −3 kg (95% CI = −1.7 to −4.2; *p* < 0.001), and TT = −3.4 kg (95% CI = −2.2 to −4.5; *p* < 0.001)), BMI (T = −0.8 kg/m^−2^ (95% CI = −0.5 to −1.2; *p* < 0.001), TP = −1 kg/m^−2^ (95% CI = −0.5 to −1.4; *p* < 0.001), and TT = −1.1 kg/m^−2^ (95% CI = −0.7 to −1.5; *p* < 0.001)), and BFP (T = −0.6% (95% CI = −0.3 to −0.9; *p* = 0.001), TP = −1.2% (95% CI = −0.8 to −1.7; *p* < 0.001), and TT = −2.1% (95% CI = −1.5 to −2.6; *p* < 0.001)). ANCOVA results showed that decreases in body mass and BMI in all three interventions were significantly greater than C while no significant differences were noted between intervention groups. Declines in BFP were significantly greater in the TT compared to all other groups, while decreases in TP were significant different than those of the C group (Table 3). No change (*p* > 0.05) was observed for nutrients intake over time (Table 4).

### 3.2. Blood Markers

**Figure 2:**
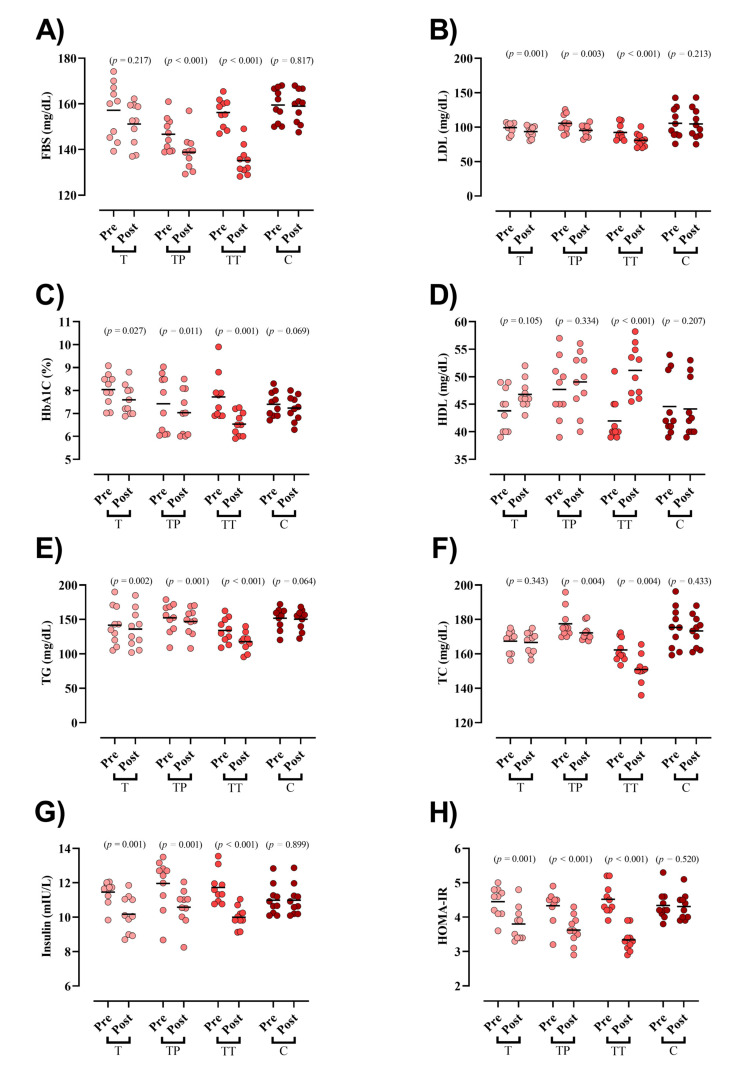
Serum markers of glucose and lipid markers. (**A**) FBS [fasting blood sugar]; (**B**) LDL [low-density lipoprotein]; (**C**) HbA1c [Hemoglobin A1c]; (**D**) HDL [high-density lipoprotein]; (**E**) TG [triglyceride]; (**F**) TC [total cholesterol]; (**G**) Insulin; and (**H**) HOMA-IR, Homeostatic Model Assessment for Insulin Resistance.

Baseline concentration of blood markers did not differ between groups except for FBS and TC. FBS was significantly reduced in both TP and TT groups (TP = −7.8 mg/dL (95% CI = −5 to −10.6; *p* < 0.001), TT = −21 mg/dL (95% CI = −15 to −27; *p* < 0.001), Figure 2A). LDL (T = −5.8 mg/dL (95% CI = −3 to −8.6; *p* = 0.001), TP = −10.4 mg/dL (95% CI = −4.6 to −16.3; *p* = 0.003), and TT = −11.4 mg/dL (95% CI = −8.9 to −13.9; *p* < 0.001), Figure 2B), HbA1c (T = −0.4 mg/dL (95% CI = −0.06 to −0.8; *p* = 0.027), TP = −0.3 mg/dL (95% CI = −0.6 to −0.1; *p* = 0.011), and TT = −1.1 mg/dL (95% CI = −0.6 to −1.7; *p* = 0.001), Figure 2C), TG (T = −5.5 mg/dL (95% CI = −2.6 to −8.3; *p* = 0.002), TP = −5.2 mg/dL (95% CI = −2.9 to −7.5; *p* = 0.001), and TT = −16.5 mg/dL (95% CI = −11.7 to −21.3; *p* < 0.001), Figure 2E), insulin (T = −1.2 mIU/L (95% CI = −0.65 to −1.91; *p* = 0.001), TP = −1.3 mIU/L (95% CI = −0.74 to −2; *p* = 0.001), and TT = −1.7 mIU/L (95% CI = −1.3 to −2; *p* < 0.001) Figure 2G), and HOMA-IR (T = −0.6 (95% CI = −0.35 to −0.94; *p* = 0.001), TP = −0.7 (95% CI = −0.43 to −0.98; *p* < 0.001), and TT = −1.1 (95% CI = −0.98 to −1.37; *p* < 0.001) Figure 2H) were significantly decreased following all interventions. HDL was significantly increased only in TT (9.1 mg/dL (95% CI = 12.7 to 5.6; *p* < 0.001), Figure 2D). Lastly, TC was significantly decreased in both TP (−5.1 mg/dL (95% CI = −2.1 to −8.2; *p* = 0.004)) and TT (−11.3 mg/dL (95% CI = −4.7 to −17.9; *p* = 0.004), Figure 2F) groups. ANCOVA results showed that the declines in FBS and LDL changes were significantly greater in the TT compared to those in the T and C groups. Moreover, the TP experienced greater drops in FBS and LDL than the C group. Changes in HbA1c, TG, HDL, and TC were significantly greater in the TT compared to all other groups. In addition, decreases in insulin concentrations were significantly greater in all experimental groups compared to C; however, no between group differences were observed between TT, TP, and T. With regard to HOMA-IR, decreases in TT were significantly greater than all other groups (Table 3).

## 4. Discussion

We examined the effects of TRX training combined with taurine supplementation on glycemic and lipid profiles as well as body composition in women with T2DM. The two main findings of our study were: (1) TRX training combined with taurine supplementation decreased HbA1c, HOMA-IR, insulin, TG, and TC and increased HDL more than all other groups, and (2) TRX training combined with taurine supplementation declined BFP to a greater extent than all other groups; however, it did not cause any synergic effects to changes in body mass and BMI seen after TRX training and taurine supplementation alone.

Although research utilizing TRX in individuals with T2D is lacking, the decreases in FBS, insulin concentrations, and insulin resistance found after TRX training with and without taurine supplementation in the present study are in agreement with a prior report in women with PCOS [21]. The number of studies on the metabolic effects of taurine supplementation in patients with T2DM is also limited. Maleki et al. evaluated the hypoglycemic and hypolipidemic effects of taurine supplementation on experimental diabetic models [31]. The findings of their study indicated that taurine supplementation (3000 mg/day) for eight weeks improved the glycemic profile by decreasing FBS, fasting insulin, and HOMA-IR as well as lipid profiles by decreasing TC and LDL in patients with T2DM. In another study, Shari et al. evaluated the effects of 1000 mg taurine supplementation for three months on glycemic control [40], which revealed no significant impact on FBS and HbA1c. The discrepancy between our results and those of Shari et al. may be related to the dose of taurine, which was 2.5 times (5 × 500 mg/d) in our study. The hypoglycemic and hypolipidemic effects of taurine are also reported in many animal studies [30,41,42]. For example, Lin et al. revealed that the treatment of taurine to T2D rats induced by a high-fat, high-sugar diet combined with Streptozotocin (STZ) injections decreased FBS, TC, TG, and HDL concentrations [43]. These findings are underlined in another study by Kim et al. in Otsuka Long-Evans Tokushima Fatty (OLETF) rats with a long-term duration of diabetes [26]. Based on their findings, taurine supplementation significantly improved glycemic and lipid profile by decreasing fasting FBS, insulin, TG, TC, HDL, and LDL. After 12 weeks, taurine supplementation significantly decreased concentrations of lipids such as TG, cholesterol, HDL, and LDL.

The effects of taurine supplementation alone and in combination with exercise on biochemical biomarkers are understudied. Only one investigation has been performed where eight weeks of taurine supplementation (1500 mg/day) combined with concurrent training decreased TC and LDL concentrations in postmenopausal women [44]. Importantly, for the first time in patients with T2DM, we have shown the synergic effects of taurine supplementation and TRX training on improving some biochemical parameters, including HbA1c, TG, TC, and HDL.

Although the main mechanisms behind the effects of the combination of taurine supplementation with exercise on the glycemic profile are unclear, the potential hypoglycemic benefits of taurine supplementation can be mediated by various mechanisms. These include decreasing hepatic glucose production by phosphorylating IRβ and hepatic Akt [45,46], reduced glucagon activity in the liver [47], enhanced insulin clearance by increase in insulin-degrading enzyme (IDE) protein [48], amplifying AMPK pathway activity in muscles [49], increased expression of the Uncoupling protein 1 (UCP1) protein in the mitochondria of adipose tissue [50,51], and improving the function, survival, and morphology of beta-pancreatic cells [52,53]. Moreover, some pathways have been suggested for hypolipidemic effects of taurine, including the augmentation of cholesterol catabolism into bile acids that can enhance the hepatic LDL depletion [54], decrease the hepatic cholesterol ester pool, improve the upregulation of LDL receptors and secretion of apolipoprotein B, and suppress LDL and Very-low-density lipoprotein (VLDL) secretion [55].

In addition, according to previous observational studies, the concentration of taurine is lower in patients with T2DM than in the general population. For example, a cross-sectional study of fifty-nine patients with T2DM and 28 healthy control participants reported that plasma taurine concentration was significantly lower in diabetic patients than healthy controls [56]. Moreover, in a 2-year randomized body mass loss dietary intervention trial, Zheng et al. revealed that baseline plasma concentration of taurine significantly interacted with the T2DM genetic predisposition on the improvement of insulin resistance [57]. They also showed that high baseline taurine concentration was associated with a lower reduction in both FBS and HOMA-IR among participants in the lowest tertile of the diabetes genetic risk score (GRS). On the contrary, a greater reduction in both insulin and HOMA-IR was reported in those participants in the highest tertile of the diabetes GRS. Moreover, Franconi et al. showed that plasma taurine concentration was lower in diabetic patients than in control participants matched for age, sex, and total and protein-derived daily energy intake [58]. Another study assessed the association of taurine concentration with insulin sensitivity among 711 overweight or obese participants during two-year dietary interventions for weight loss. The results showed that higher taurine concentration could modulate the diabetes-related genes and HOMA-IR [57]. Similarly, we observed positive effects of taurine supplementation on HbA1c in patients with T2DM.

Regarding body composition, in this study, all three interventions showed a significant decrease in body mass, BMI, and BFP. Importantly, TRX training with taurine supplementation induced greater reductions in BFP compared to all other groups. Contrary to our findings, taurine (3 g/day) did not induce additional changes in body composition during an 8-week dietary weight-loss period in females with obesity [59]. Some recent studies underlined the positive effects of taurine on fat oxidation. For example, Carvalho et al. showed that 6 g of taurine supplementation 90 min before a single bout of aerobic exercise might increase the lipid oxidation post-exercise in healthy young men [60]. Moreover, it has been shown that taurine supplementation associated with exercise improved lipid metabolism by modulating genes related to mitochondrial activity and fatty acid oxidation in the subcutaneous white adipose tissue of obese women [61]. In an animal study, Martiniano et al. showed that taurine supplementation with exercise training (30 to 60 min treadmill running for four days at the speed of 5 to 10 m/min) was able to reduce visceral fat area and weight compared to exercise alone in obese rats after eight weeks [62]. Regarding TRX training, there is some evidence that indicates no significant differences in body composition changes between TRX training and traditional RT [20,63]. TRX, as an RT modality, can improve body fat through different mechanisms such as increasing daily energy expenditure, decreasing appetite (by suppressing orexigenic hormones and stimulating the anorexigenic hormones), and stimulating growth hormone secretion [64,65,66].

The strengths of this investigation include the use of four different groups, which afforded direct comparisons between TRX training, taurine supplementation, and their combination. Further strengths include the inclusion of sedentary middle-aged T2D women, high training and supplementation compliance rates, and TRX protocols prescribed according to prior research. A limitation of our study is the utilization of skinfold measurements to calculate body composition. Although this technique is not as accurate as dual-energy X-ray absorptiometry (the gold standard technique), previous studies have shown that it is a valid and reliable method [67,68] that appears to be the least affected by factors that are difficult to control in research, such as food intake, hydration status, and daily activity [69]. Moreover, the 3-site skinfold Jackson–Pollock equation [33] used in our study has received thousands of citations, indicating that, despite the limitations of skinfold measurements, it is a method widely utilized in research.

## 5. Conclusions

In conclusion, TRX alone improved glycemic and lipid profile in middle-aged T2D women. Notably, the synergic effects of TRX training and taurine supplementation were shown in HbA1c, HOMA-IR, insulin, TG, TC, HDL, and BFP changes. Moreover, TRX training, taurine supplementation, and combination of these two interventions declined body mass and BMI without any synergic effects. Because the combined treatment concomitantly improved glycemic and lipid profiles and BFP in our medicated participants, present outcomes suggest that TRX training + taurine supplementation may be an effective adjuvant therapy in individuals with T2D.

## Figures and Tables

**Table 1:** Descriptive characteristics of participants (mean ± SD).

	T	TP	TT	C
Age (y)	53.3 ± 3.8	55 ± 5.7	49.4 ± 6.3	54.4 ± 3.7
Body mass (kg)	86.1 ± 5	84.5 ± 4.6	83 ± 5.3	83.6 ± 5.7
BMI (kg·m^−2^)	27.9 ± 2.1	28.9 ± 2.5	27.6 ± 1.8	27 ± 1.6
BFP (%)	29.1 ± 2.4	30.5 ± 2.5	29.3 ± 3.3	30.3 ± 2.9
FBS (mg/dL)	157.2 ± 12.3	146.7 ± 7.4	156.2 ± 6.2	159.4 ± 7.3
HbA1C (%)	8 ± 0.6	7.4 ± 1.2	7.7 ± 0.9	7.3 ± 0.5
HDL (mg/dL)	43.8 ± 3.9	47.7 ± 5.5	42 ± 3.8	44.5 ± 5.6
LDL mg/dL)	99.4 ± 7.7	105.6 ± 12.6	92.2 ± 11.4	105.5 ± 21.8
TG (mg/dL)	141.6 ± 27.7	152.2 ± 21.4	134.2 ± 17.8	151.8 ± 15.5
TC (mg/dL)	167.4 ± 6.3	177.4 ± 8.5	162.2 ± 6.6	175.2 ± 12.1
Insulin (mIU/L)	11.4 ± 0.6	11.9 ± 1.4	11.7 ± 0.9	10.9 ± 0.8
HOMA-IR	4.4 ± 0.4	4.3 ± 0.4	4.5 ± 0.4	4.3 ± 0.4

**Abbreviations:** T, taurine supplementation; TP, TRX suspension training + placebo; TT, TRX suspension training + taurine supplementation; C, control; BMI, body mass index; BFP, body fat percentage; FBS, fasting blood sugar; HbA1c, hemoglobin A1c; HDL, high density lipoprotein; LDL, low-density lipoprotein; TG, Triglyceride; TC, total cholesterol; HOMA-IR, Homeostatic Model Assessment for Insulin Resistance.

**Table 2:** RPE and repetitions. Abbreviations. RPE, Borg Rating of Perceived Exertion.

Week	RPE	Repetition
1–2	14	12
3–4	14–15	12
5–6	15–16	10
7–8	16–17	8

**Table 3:** The effect of group on the post-time markers controlled by the pre-time markers using ANCOVA.

Variable	Contrast	β (Std. Error)	95% CI	*p*-Value
body mass-post	T vs. C	−3.10 (0.67)	−4.98, −1.22	<0.001
	TP vs. C	−3.40 (0.66)	−5.26, −1.54	<0.001
	TT vs. C	−3.80 (0.66)	−5.66, −1.95	<0.001
	T vs. TP	0.30 (0.67)	−1.57, 2.17	1.000
	T vs. TT	0.70 (0.68)	−1.20, 2.60	1.000
	TP vs. TT	0.40 (0.67)	−1.46, 2.27	1.000
BMI-post	T vs. C	−1.04 (0.22)	−1.64, −0.44	<0.001
	TP vs. C	−1.22 (0.23)	−1.85, −0.59	<0.001
	TT vs. C	−1.28 (0.21)	−1.88, −0.68	<0.001
	T vs. TP	0.18 (0.22)	−0.43, 0.78	1.000
	T vs. TT	0.24 (0.21)	−0.36, 0.84	1.000
	TP vs. TT	0.06 (0.22)	−0.55, 0.67	1.000
BFP-post	T vs. C	−0.70 (0.25)	−1.39, −0.02	0.043
	TP vs. C	−1.26 (0.24)	−1.95, −0.58	<0.001
	TT vs. C	−2.15 (0.25)	−2.84, −1.46	<0.001
	T vs. TP	0.56 (0.25)	−0.13, 1.25	0.181
	T vs. TT	1.44 (0.24)	0.76, 2.13	<0.001
	TP vs. TT	0.88 (0.25)	0.20, 1.57	0.006
FBS-post	T vs. C	−6.97 (3.28)	−16.13, 2.20	0.244
	TP vs. C	−15.63 (3.72)	−26.03, −5.23	0.001
	TT vs. C	−22.66 (3.29)	−31.86, −13.45	<0.001
	T vs. TP	8.67 (3.58)	−1.34, 18.68	0.125
	T vs. TT	15.69 (3.27)	6.56, 24.82	<0.001
	TP vs. TT	7.02 (3.53)	−2.84, 16.88	0.325
HbA1C-post	T vs. C	−0.05 (0.19)	−0.57, 0.48	1.000
	TP vs. C	−0.21 (0.18)	−0.71, 0.29	1.000
	TT vs. C	−0.897 (0.18)	−1.41, −0.39	<0.001
	T vs. TP	0.17 (0.19)	−0.35, 0.68	1.000
	T vs. TT	0.85 (0.18)	0.35, 1.36	<0.001
	TP vs. TT	0.69 (0.18)	0.18, 1.19	0.003
HOMA-IR	T vs. C	−0.58 (0.13)	−0.94, −0.22	<0.001
	TP vs. C	−0.68 (0.13)	−1, −0.32	<0.001
	TT vs. C	−1 (0.13)	−1.46, −0.72	<0.001
	T vs. TP	0.098 (0.13)	−0.26, 0.46	1.000
	T vs. TT	0.5 (0.13)	0.14, 0.87	0.002
	TP vs. TT	0.41 (0.13)	0.04, 0.77	0.021
HDL-post	T vs. C	3.11 (1.67)	−1.56, 7.78	0.428
	TP vs. C	3.15 (1.71)	−1.65, 7.95	0.450
	TT vs. C	8.50 (1.70)	3.74, 13.26	<0.001
	T vs. TP	−0.04 (1.74)	−4.91, 4.83	1.000
	T vs. TT	−5.39 (1.68)	−10.11, −0.68	0.017
	TP vs. TT	−5.35 (1.82)	−10.45, −0.24	0.035
TG-post	T vs. C	−4.99 (1.80)	−10.03, 0.05	0.054
	TP vs. C	−3.72 (1.77)	−8.68, 1.24	0.260
	TT vs. C	−16.71 (1.86)	−21.90, −11.52	<0.001
	T vs. TP	−1.27 (1.80)	−6.32, 3.78	1.000
	T vs. TT	11.72 (1.79)	6.72, 16.72	<0.001
	TP vs. TT	12.99 (1.86)	7.79, 18.19	<0.001
TC-post	T vs. C	−2.14 (2.51)	−9.16, 4.88	1.000
	TP vs. C	−2.34 (2.39)	−9.03, 4.35	1.000
	TT vs. C	−14.94 (2.72)	−22.55, −7.32	<0.001
	T vs. TP	0.20 (2.59)	−7.04, 7.44	1.000
	T vs. TT	12.80 (2.44)	5.98, 19.62	<0.001
	TP vs. TT	12.60 (2.83)	4.67, 20.52	0.001
LDL-post	T vs. C	−5.84 (2.10)	−11.74, 0.061	0.054
	TP vs. C	−9.64 (2.08)	−15.47, −3.82	<0.001
	TT vs. C	−12.38 (2.20)	−18.54, −6.21	<0.001
	T vs. TP	3.80 (2.11)	−2.09, 9.72	0.479
	T vs. TT	6.54 (2.12)	0.61, 12.47	0.024
	TP vs. TT	2.73 (2.20)	−3.43, 8.90	1.000
Insulin-post	T vs. C	−1.14 (0.27)	−1.89, −0.38	0.001
	TP vs. C	−1.07 (0.28)	−1.86, −0.28	0.003
	TT vs. C	−1.46 (0.27)	−2.27, −0.72	<0.001
	T vs. TP	−0.06 (0.27)	−0.82, 0.69	1.000
	T vs. TT	0.35 (0.26)	−0.39, 1.10	1.000
	TP vs. TT	0.42 (0.26)	−0.32, 1.17	0.751

**Abbreviations**: BMI, body mass index; BFP, body fat percentage; FBS, fasting blood sugar; HbA1c, hemoglobin A1c; LDL, low-density lipoprotein; TC, total cholesterol; TG, Triglyceride; HDL, high density lipoprotein; HOMA-IR, Homeostatic Model Assessment for Insulin Resistance; T, taurine supplementation; TP, TRX suspension training + placebo; TT, TRX suspension training + taurine supplementation; C, control.

**Table 4:** Energy and macronutrients at the baseline and at the end of 8 weeks.

Variables	Group	Pre-Test	Post-Test	*p*-Value
Energy intake (kcal/day)	T	2038 ± 285.2	1940.9 ± 263.8	0.434
	TP	1954.9 ± 193	1904.3 ± 216.7	0.617
	TT	1945.3 ± 264.1	1858.6 ± 287.4	0.522
	C	1962.4 ± 238.8	2004.1 ± 338.1	0.433
Carbohydrate (g/day)	T	235.8 ± 41.1	224.9 ± 38.7	0.392
	TP	221.9 ± 31.6	219.4 ± 41.8	0.878
	TT	219.7 ± 35.1	212.5 ± 43.9	0.697
	C	228 ± 23.3	233.8 ± 38.5	0.560
Protein (g/day)	T	67.4 ± 14	66 ± 13.1	0.689
	TP	66.9 ± 12.4	63.4 ± 13.7	0.310
	TT	63.6 ± 13.3	59.8 ± 10.7	0.532
	C	64.4 ± 13.4	65 ± 14.3	0.853
Fat (g/day)	T	91.6 ± 20.1	86.2 ± 20.1	0.580
	TP	88.8 ± 13.9	85.8 ± 13.4	0.671
	TT	90.2 ± 13.1	85.4 ± 18.1	0.486
	C	88 ± 19.7	89.8 ± 21.1	0.624

**Abbreviations:** T, taurine supplementation; TP, TRX suspension training + placebo; TT, TRX suspension training + taurine supplementation; C, control.

## Data Availability

The data presented in this study are available on request from the corresponding authors.
